# Laser Curve Extraction of Wheelset Based on Deep Learning Skeleton Extraction Network

**DOI:** 10.3390/s22030859

**Published:** 2022-01-23

**Authors:** Shuai Luo, Kai Yang, Lijuan Yang, Yong Wang, Xiaorong Gao, Tianci Jiang, Chunjiang Li

**Affiliations:** 1 School of Physical Science and Technology, Southwest Jiaotong University, Chengdu 610031, China; yeluo@my.swjtu.edu.cn (S.L.); wangyonga@swjtu.edu.cn (Y.W.); gxrr@home.swjtu.edu.cn (X.G.); 2 School of Mathematics, Sichuan Normal University, Chengdu 610066, China; 20200892014@stu.sicnu.edu.cn; 3 School of Mechanical Engineering, Waseda University, Kitakyushu 8080135, Japan; jiangtianci@ruri.waseda.jp; 4 School of Mechanics, Zhejiang University, Hangzhou 310058, China; lcj@zju.edu.cn

**Keywords:** deep learning, semantic segmentation, laser curve extraction, image processing

## Abstract

In this paper, a new algorithm for extracting the laser fringe center is proposed. Based on a deep learning skeleton extraction network, the laser stripe center can be extracted quickly and accurately. Skeleton extraction is the process of reducing the shape image to its approximate central axis representation while maintaining the image’s topological and geometric shape. Skeleton extraction is an important step in topological and geometric shape analysis. According to the characteristics of the wheelset laser curve dataset, a new skeleton extraction network, a hierarchical skeleton network (LuoNet), is proposed. The proposed architecture has three levels of the encoder–decoder network, and YE Module interconnection is designed between each level of the encoder and decoder network. In the wheelset laser curve dataset, the F1_score can reach 0.714. Compared with the traditional laser curve center extraction algorithm, the proposed LuoNet algorithm has the advantages of short running time, high accuracy, and stable extraction results.

## 1. Introduction

In recent years, deep learning has made remarkable progress in the three main fields of computer vision image recognition, target detection, and image segmentation. While deep learning approaches are comparable to human vision in many areas, some areas require designing different models for different tasks. In this paper, a new skeleton extraction method based on deep learning is proposed to extract the laser curve of a subway wheelset.

Skeletonization is the process of extracting or generating an approximate geometric representation of a shape (skeleton) with the aim of reducing it to clean skeleton pixels to preserve the range and connectivity of the original shape. Skeleton extraction combines local and global knowledge of shapes. The skeleton is a compact and intuitive central axis representation of the shape, which preserves the topology and geometry of the shape. Skeleton representations of shapes can be used for a variety of purposes, such as modeling, manipulation, composition, matching, registration, compression, and analysis.

At present, the laser stripe center extraction of a subway wheelset mainly relies on the traditional algorithm, which has low efficiency and low accuracy. The gray center of gravity method [1], curve fitting method [2], and extreme value method [3] often require that the gray value of the laser fringe be distributed into an ideal Gaussian distribution, but the obtained fringe is not an ideal Gaussian distribution and is easily disturbed by noise. The template matching method is limited by the limited template direction, and the accuracy of fringe center extraction is affected by the surface roughness of the object [4]. The Steger method has a large amount of computation, low efficiency, and the improper selection of a Gaussian kernel will lead to image information distortion [5]. Due to the influence of light, the stripe center line extracted by Zhang-Suen method has burrs, which increases the width by more than one pixel [6]. The ridge tracking method and threshold method are very sensitive to noise and easy to be disturbed by noise [7]. Although many scholars have tried to introduce deep learning methods, most of them are used for denoising and cannot directly extract the center of the laser stripe. For example, Wang Shengchun et al. [8] used deep learning E-Net for preprocessing, followed by template matching, and the gray gravity center method for subsequent processing, and Yang Kai et al. [9] used UNet network for laser fringe segmentation. The results of these methods often depend on the effect of deep learning preprocessing and consume a great deal of time.

The goal of the task of skeleton segmentation is to extract the skeleton with the desired width of one pixel and keep the topology and geometry of the shape consistent with the target extracted from the laser stripe center. We take the skeleton extraction problem of subway wheelset laser curves as a segmentation task where semantic segmentation networks learn to classify shaped pixels as skeleton pixels or backgrounds. By designing a skeleton extraction network (LuoNet) to extract the center of the laser stripe, the extraction accuracy of the algorithm can be effectively improved by using the YE Module. The center of the laser fringe can be extracted quickly and accurately by the skeleton extraction network.

## 2. Related Work

Deep learning skeleton extraction is an emerging field, and skeleton extraction of the network has sprung up in recent years. With continuously more accurate skeleton extracted datasets, deep learning skeleton extraction is becoming more and more sought after by researchers, but the development is still limited by the network structure skeleton extraction, so skeleton extraction accuracy still has a long way to go.

In 2019, Demir et al. [10] defined the task of skeletonization as the use of conditional adversative neural networks to transform pre-segmented images into bones. Using distance transformation to preprocess binary input shapes, they found that this preprocessing of input data significantly improved the learning ability of neural networks. A normal pixel2pixel model is then used to extract the skeleton. This model achieved an F1-score of 0.6244 on the Pixel SkelNetOn validation dataset.

Jiang et al. [11] constructed a full convolutional network named Feature Hourglass Network (FHN) for skeleton detection in 2019. FHN takes advantage of the rich characteristics of a fully convolutional network and integrates side outputs in a hierarchical way from deep to shallow layers to reduce the residual between prediction results and ground truth. This model achieved an F_score of 0.6325 on the Pixel SkelNetOn validation dataset.

Nathan et al. [12] proposed the unique U-Net architecture in 2019, which integrates the HED architecture into the decoding process of U-Net. They used four side layers fused to one dilated convolution layer for increased performance. Their model achieved an F_score of 0.7480 on the Pixel SkelNetOn validation dataset.

In 2019, Panichev et al. [13] proposed a unique U-Net network whose main idea is to edit skeleton masks uniformly through different scaling layers through feature propagation. It is opposed to ultimately generating bones through representations of object shapes at different scales, which are extracted from real images through depth supervision. The model achieved an F_score of 0.7500 on the Pixel SkelNetOn validation dataset.

Dey et al. [14] proposed a sub-pixel dense thinning network in 2020, which uses a three-level encoder–decoder network with the dense interconnection between decoder networks at each level. In this structure, sub-pixel convolution is used to replace the general upsampling layer and transpose the convolution layer to minimize the information loss during the upsampling period.

## 3. Materials and Methods

In the field of laser curve extraction, most of the traditional methods, such as gray distribution or geometric relation, are used to extract the center of the stripe. The traditional method takes a great deal of time, and the time cost is exchanged for accurate improvement. In a real-world scenario, this is not good. In recent years, although many scholars have introduced deep learning, most of them are introduced in the form of data preprocessing, without giving full play to the advantages of deep learning. In this paper, the LuoNet network is proposed to extract the center of the light stripe of train wheelset tread by using the unique advantages of the neural network. Great improvements have been made in time and accuracy.

### 3.1. Experimental Dataset

**Figure 1:**
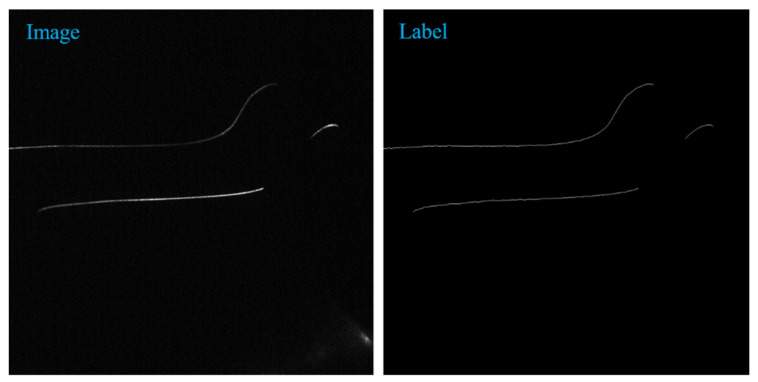
Laser curve image and label of subway wheelset.

The dataset used in this experiment comes from Chengdu Metro and is provided by Chengdu Leading Technology Co., Ltd in Chengdu, China. It contains various photos of subway operation, including fatigue, abrasions, wear, cracks, dents, and so on. Due to the dark subway driving environment, the gray value of the collected photos is low, so it is difficult to extract the center of the light stripe accurately and effectively by traditional methods. The dataset of subway wheelset laser curve contains 100 original pictures with the size of 1236 pixels * 1624 pixels and corresponding annotated pictures. Figure 1 is the original image and labeled image of the subway wheelset data graph. The left is the original image, and the right is the labeled image. In data annotation, per-pixel annotation is adopted to make the center of the light stripe fall on the annotation point as far as possible, so the label is a thin line of one-pixel width.

### 3.2. Data Augmentation

**Figure 2:**
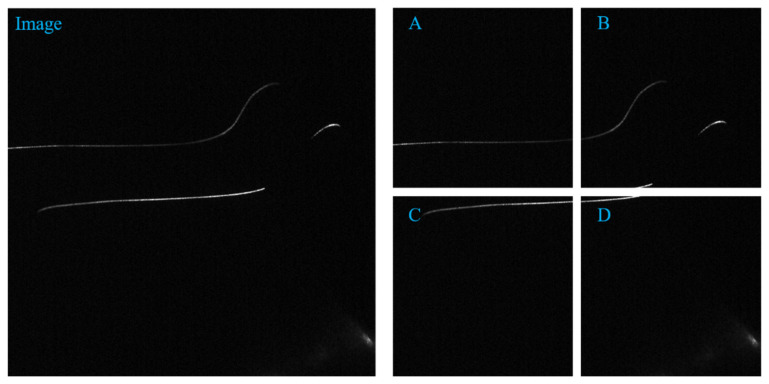
The original image and its clipping ((**A**–**D**) are the clipped images).

**Figure 3:**
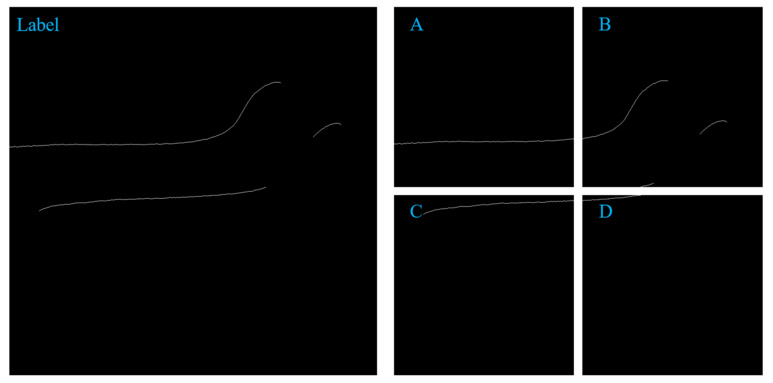
The original label and its cut image ((**A**–**D**) are the clipped images).

Through the segmentation and cutting technology, an original image of 1236 pixels * 1624 pixels and a label image can be cut and segmented to 4 images of 512 * 512 size. as shown in Figure 2 and Figure 3 below. The expanded dataset contains 400 images with 512 * 512 pixels and labeled images. At the same time, images with fewer than 500 pixels in the image are discarded. The segmentation and cutting of the picture can increase the proportion of the foreground in the picture, help solve the detailed problem, and help the convergence of the model.

**Figure 4:**
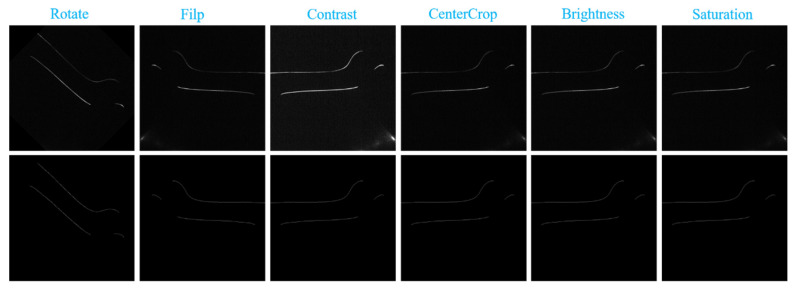
Data enhancement.

In deep learning, when the amount of data is insufficient, data expansion technology is often used to expand the data in order to train a good network. In this paper, the train wheelset fringe dataset is expanded by multi-angle rotation and mirror mapping. As shown in Figure 4, the original image and annotated image can expand the dataset by 7 times after being flipped from different angles, such as up and down, left and right, and diagonal.

### 3.3. The Neural Network Architecture

**Figure 5:**
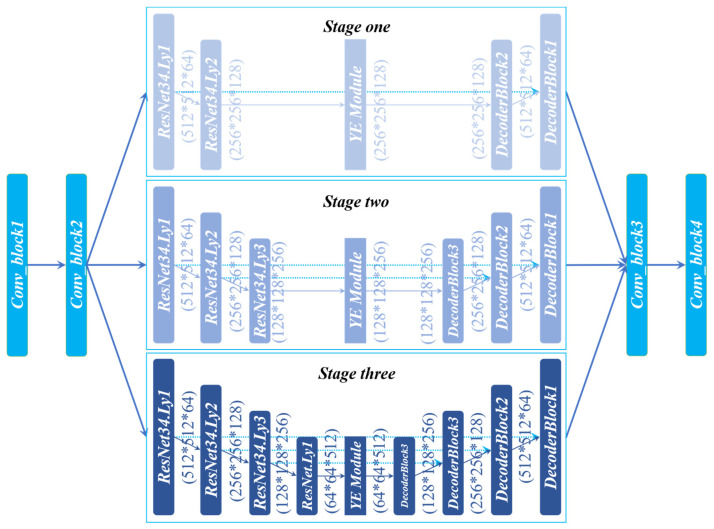
LuoNet network structure.

The skeleton extraction network (LuoNet) designed by us is shown in Figure 5. The whole network can be regarded as composed of seven encoding–decoding networks. The network is mainly divided into three parts: left module, middle module, and right module. The left part side is two convolution modules, and each convolution layer contains convolution, regularization, and activation functions. The middle part is divided into three stages, namely Stage One, Stage Two, and Stage Three. Each stage contains a U-Net network structure of different depth, and the depth of the three U-Nets from top to bottom is increased accordingly. Based on the characteristics of the dataset, the data of each image can be divided into three categories according to the proportion of foreground and background. The feature images with a small proportion of foreground and background can be passed through Stage One because the depth of the Stage One network is shallow and the number of Maxpool is small, so it can provide good detailed information. The feature map with a medium proportion of foreground and background is passed in Stage Two. As the network layer number gradually deepens, the number of Maxpool increases, the receptive field of the network increases, and the extracted high-level features increase. When the feature map with the largest proportion of foreground and background reaches Stage Three, the network depth is the largest and the abstract features extracted are the most. In Stage One, there are many low-dimensional feature graphs, so semantic information is less and detailed information is rich. The semantic information and detailed information contained in Stage Two are at a medium level. In Stage Three, there are more high-dimensional feature graphs with rich semantic information and less detailed information. The feature map output by these three stages will make a concat and then input it to the right part, which contains two convolutional layer modules. The whole network structure has the following three characteristics. It realizes the fusion of high-dimensional feature graphs and low-dimensional feature graphs and the fusion of detailed information and semantic information. The width of the network is increased, and the receptive field of the network is improved. In a cascading way, the loss caused by subsampling is minimized.

### 3.4. UNet_A

**Figure 6:**
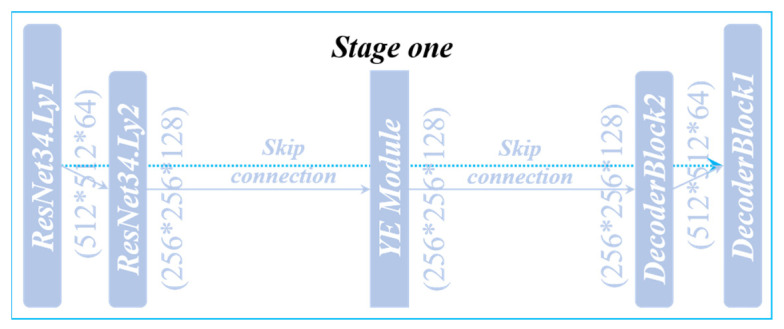
UNet_A structure.

The UNet_A network structure (UNet_A) used in Stage One is shown in Figure 6. ResNet34 is adopted as the backbone in the coding part, which can enhance the transmission of features and reduce the number of parameters. The attention module (YE Module) is added at the bottom of the network structure. In the UNet_A Module, more shallow features can be paid attention to through YE Module so that Stage One can learn more detailed information and help the decoding process to better restore features. The decoding part is composed of DecoderBlock, which can decode information, recover spatial information, and improve the resolution. The depth of the whole structure is divided into two layers, the number of channels in the coding layer increases from 64 to 128, and the size of the feature map decreases from 512 * 512 to 256 * 256. After the YE Module, it enters the decoding stage, the number of channels is reduced from 128 to 64 again, and the size of the feature map is restored from 256 * 256 to 512 * 512. Each layer makes up for the loss caused by Encoder through Skip Connection. When a network user uses only UNet_A, the model is called LuoNet_A.

### 3.5. UNet_B

**Figure 7:**
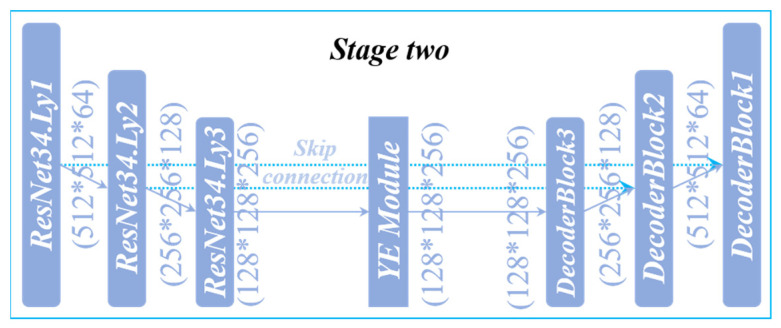
UNet_B structure.

In Stage Two, the UNet_B network structure (UNet_B) is used, as shown in Figure 7. ResNet34 is used as the backbone in the coding structure, which can strengthen the transmission of features and reduce the number of parameters. The attention module (YE Module) is added at the bottom of the network structure. In UNet_B, the shallow and deep features can be paid attention to through YE Module, which enables the network to obtain more detailed information and spatial information. The decoding part is composed of DecoderBlock with a small number of parameters, which can decode information, recover spatial information, and improve the resolution. The depth of the whole structure is three layers. The number of channels in the coding layer increases from 64 to 128 and then from 128 to 256. The size of the feature map decreases from 512 * 512 to 256 * 256, and then from 256 * 256 to 128 * 128. After the attention module, the stage of decoding is entered, channel number again goes from 256 to 128, down from 128 to 64, the future map size is restored from 128 * 128 to 256 * 256, and then from 256 * 256 to 512 * 512, each layer of the encoding and decoding layer through the Skip Connection is linked together, can reduce the coding layer because of the loss caused by the Downsampling, and, at the same time, help the Decoder to recover more detailed information. When a network uses only UNet_B, the model is called LuoNet_B.

### 3.6. UNet_C

**Figure 8:**
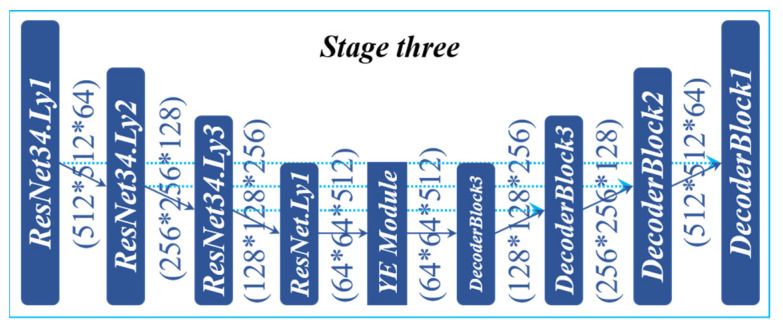
UNet_C structure.

The UNet_C network structure (UNet_C) used in Stage Three is shown in Figure 8. ResNet34 is used as the backbone in the part of the coding structure, which can enhance the transmission of the features and reduce the number of parameters. The attention module (YE Module) is added at the bottom of the network structure. In UNet_C, more abstract features can be learned through YE Module, which is conducive to better classification. The decoding stage is composed of DecoderBlock, which has fewer parameters and can restore spatial information to improve the resolution of the feature map. The depth of the whole structure is four layers. The number of channels of the encoding stage increases from 64 to 128, and then from 128 to 256, and then from 256 to 512. The size of the future map decreases from 512 * 512 to 256 * 256, and then from 256 * 256 to 128 * 128, and then from 128 * 128 to 64 * 64. After the attention module, the size and channel of the future map remain unchanged. Then, it enters the decoding stage. The channel is reduced from 512 to 256 again, and then from 256 to 128, and then from 128 to 64 again. The coding layer and decoding layer of each layer are connected through Skip Connection to reduce the loss of the coding layer due to the downsampling and help the Decoder to recover more detailed information. When a network uses only UNet_C, the model is called LuoNet_C.

### 3.7. The Attention Module

**Figure 9:**
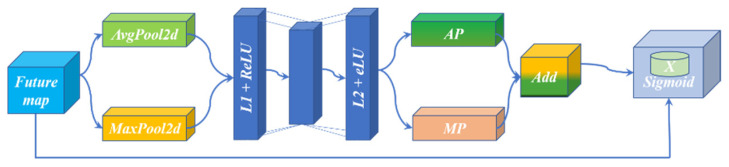
YE Module.

The attention block (YE Module) is designed by using the idea of the SENet [15] network to improve the attention to the target area and better extract the skeleton. As shown in Figure 9, YE Module consists of two paths: Avgpooling Way and Maxpooling Way, both of which pass through two linear layers, each of which is followed by different activation functions. The activation function at the L_1_ layer is ReLU, and the activation function at the L_2_ layer is eLU. When entering the linear layer, the channels will be reduced first and then returned to realize feature enhancement. Then, the results of the two paths are further added together to strengthen the features that need to be prominent again, and the double enhancement of the features is realized through two intensifications. The original input is then multiplied and processed by the activation function sigmoid.

(1) $$M(f)=\theta (L_{2}(L_{1}(\mathrm{AvgPool}(f)))+L_{2}(L_{1}(\mathrm{MaxPool}(f))))$$

In Formula (1), after feature graph *f* enters the AvgPool layer, it enters linear layer L_1_ and linear layer L_2_ to obtain feature graph AP. After feature graph *f* enters the MaxPool layer, it also enters linear layer L_1_ and linear layer L_2_ to obtain feature graph MP. Finally, a sigmoid operation is performed on the result of adding AP and MP.

### 3.8. DecoderBlock

**Figure 10:**
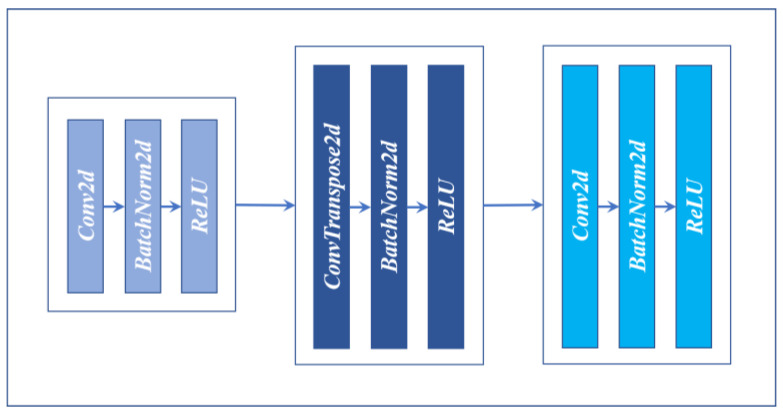
DecoderBlock.

The encoding structure (DecoderBlock) is shown in Figure 10. DecoderBlock has three convolution layers. The first layer is made up of a convolution of Kernel Size 1, Strides 1, including the BN layer and ReLU layer. The second layer is made up of the Deconvolution of Kernel Size 3, Strides 2, and includes the BN layer and ReLU layer. The third layer, like the first one, contains Kernel Size 1, Strides 1, Convolution, BN, ReLU, with the difference in channels for input and output. The whole structure channel is first decreased and then increased, similar to an hourglass. The purpose of this method is not only to reduce the number of parameters but also to recover spatial information better.

### 3.9. Evaluation Metric

The skeleton extraction task can be regarded as a semantic segmentation task with higher requirements. Therefore, evaluation indexes commonly used in semantic segmentation tasks, such as Miou, Precision, Recall, and F_Score, can be used. They can pay more attention to where the skeleton appears in the target and evaluate our results better. The number of skeleton pixels (positive) or background pixels (negative) is first counted in both the generated and ground truth skeleton images. The F1_score is then calculated from the harmonic average of the precision and recall values as follows:

(2) $$F1\_\mathrm{score}=\frac{2\times \mathrm{precision}\times \mathrm{recall}}{\mathrm{precision}+\mathrm{recall}}$$

(3) $$\mathrm{Precision}=\frac{\mathrm{TP}}{\mathrm{TP}+\mathrm{FP}}$$

(4) $$\mathrm{Recall}=\frac{\mathrm{TP}}{\mathrm{TP}+\mathrm{FN}}$$

(5) $$\mathrm{Miou}=\frac{\mathrm{TP}}{\mathrm{TP}+\mathrm{FP}+\mathrm{FN}}$$

where TP, FN, and FP stand for several pixels for true positives, false negatives, and false positives, respectively. Precision is the percentage of correctly predicted skeleton pixels in all predicted skeleton pixels, while recall is the percentage of correctly predicted skeleton pixels in all true skeleton pixels. The comprehensive index F1 value integrates precision and recall, and the contradiction between them is balanced by calculating the weighted and the harmonic average of the two. The intersection ratio IOU is the ratio of the intersection and the union of the target pixels predicted by the model and the real target pixels in reality, and it is a common evaluation index in segmentation and detection studies.

## 4. Experimental Evaluation

**Figure 11:**
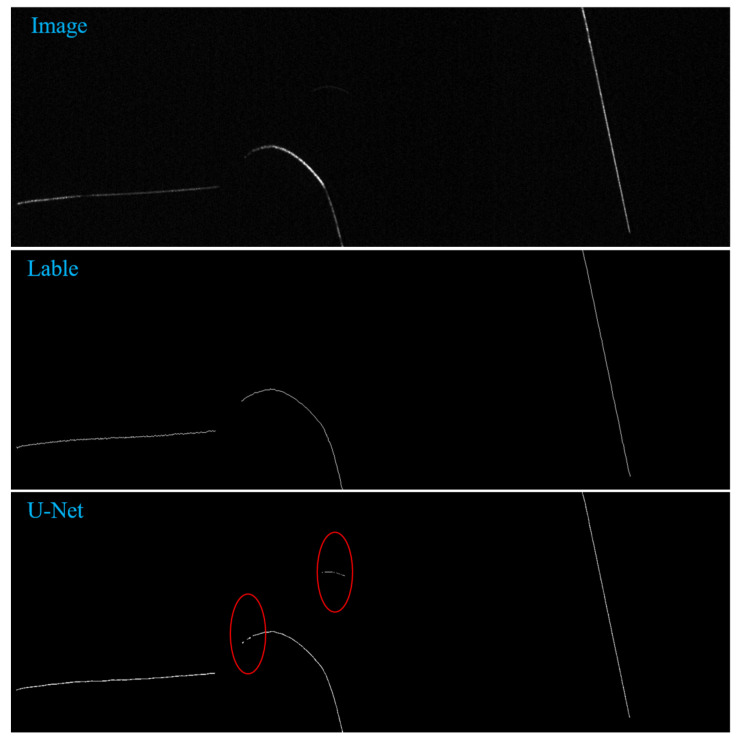

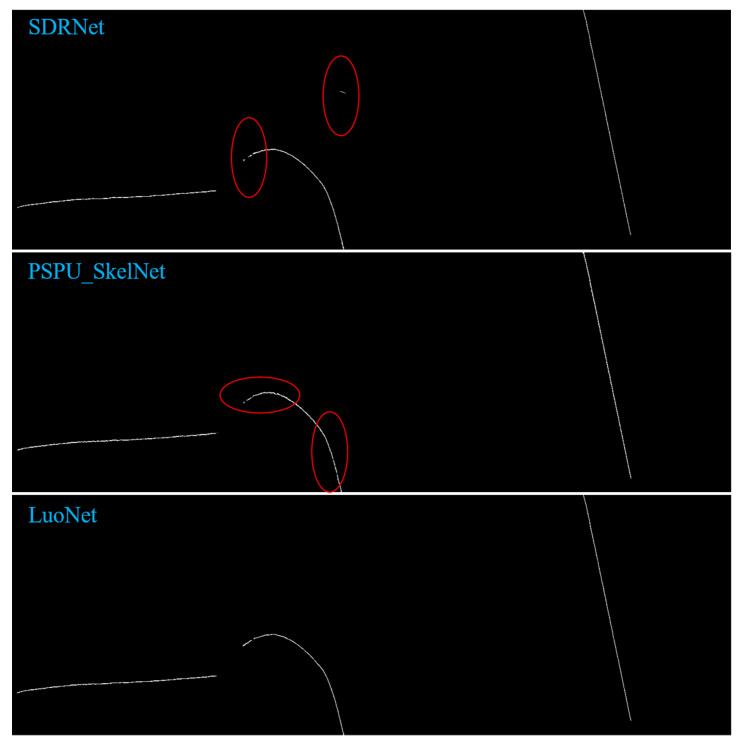
Visualization results of different networks. (From top to bottom: Images, Ground Truth Skeleton, UNet Predicted Skeleton, SDRNetPredicted Skeleton, PSPU_SkelNet Predicted Skeleton, LuoNet Predicted Skeleton).

We trained LuoNet for 200 epochs starting with a learning rate of 1 × 10^−3^ and decreasing to 1 × 10^−4^ at 60 epochs and 1 × 10^−5^ at 120 epochs. Adam optimizer is used for training, the learning rate is set with the distribution decline method, and the Loss function adopts the Binary cross-entropy and Dice Loss. To verify the effectiveness of the network extraction results designed in this paper, we used horizontal, curved, and vertical samples to test, and compared the recently popular skeleton extraction networks. As shown in Figure 11, we have visualized the extraction results of different networks from top to bottom, namely the skeleton extraction results of UNet [16], SDRNet [17], PSPU_SkelNet, and LuoNet. By comparing the original image and label image with the extraction results of UNet and SDRNet, we can find that the extraction results of UNet and SDRNet identify the places that are not skeletons as skeletons, and the places with relatively small gray value have more serious losses. From the comparison of PSPU_SkelNet with the original image and label image, the extraction results of PSPU_SkelNet can distinguish the real skeleton to a certain extent, but the places with low gray values also have some missing parts. Compared with the extraction results of other networks, the LuoNet designed by us can achieve a stable effect even in the place where the gray value is relatively low. The LuoNet designed by us can achieve a good effect no matter if in the horizontal, curved, and longitudinal samples.

To better compare, in this paper, the evaluation generated data visualization results, as shown in Table 1. By comparing F1_score, Recall, Precision and Miou indexes, it can be seen that the extraction results of LuoNet indexes proposed in this paper are better than those of other networks. Therefore, using LuoNet to extract the laser curves of subway wheelsets can obtain better results.

YE Module Ablation Study: In order to prove the effectiveness of the YE Module proposed in this paper, this paper makes a comparison with the SE attention module proposed by SENet. In order to better reflect the usefulness of the YE Module proposed in this paper, a comparative study was conducted in this paper to evaluate the effectiveness of the attention module proposed in this paper from the four indexes of F1_score, Precision, Recall, and Miou. As can be seen from the Table 2 ablation experiment, the F1_score, Precision, Recall, and Miou of the LuoNet + YE prediction results reached 71.4%, 62.3%, 83.5%, and 78.1%, respectively. The four indexes were all higher than those of the LuoNet + SE group and LuoNet group. It is effectively proved that the YE Module proposed in this paper can help the model to better extract the stripe center of the laser curve of the subway wheelset.

Models Ablation Study: A model ablation experiment was also conducted in this paper. Data tests were conducted on the visualization results of LuoNet_A, LuoNet_B, LuoNet_C, LuoNet_AB, LuoNet_BC, LuoNet_CA, and LuoNet (LuoNet_ABC). The test results are shown in Table 3, and it can be seen from the data indicators that the LuoNet designed by us has the best effect. The F1_score, Accuracy rate, Recall rate, and Miou ratio of LuoNet’s prediction results reached 71.4%, 62.3%, 83.5%, and 78.1%, respectively. The four indexes were all higher than the experimental results of LuoNet_A, LuoNet_B, LuoNet_C, LuoNet_AB, LuoNet_BC, and LuoNet_CA groups. It can be seen that LuoNet can better extract the stripe center of a subway wheelset laser curve, so we chose LuoNet as the subway wheelset laser curve centerline extraction algorithm.

## 5. Light Stripe Center Extraction

**Figure 12:**
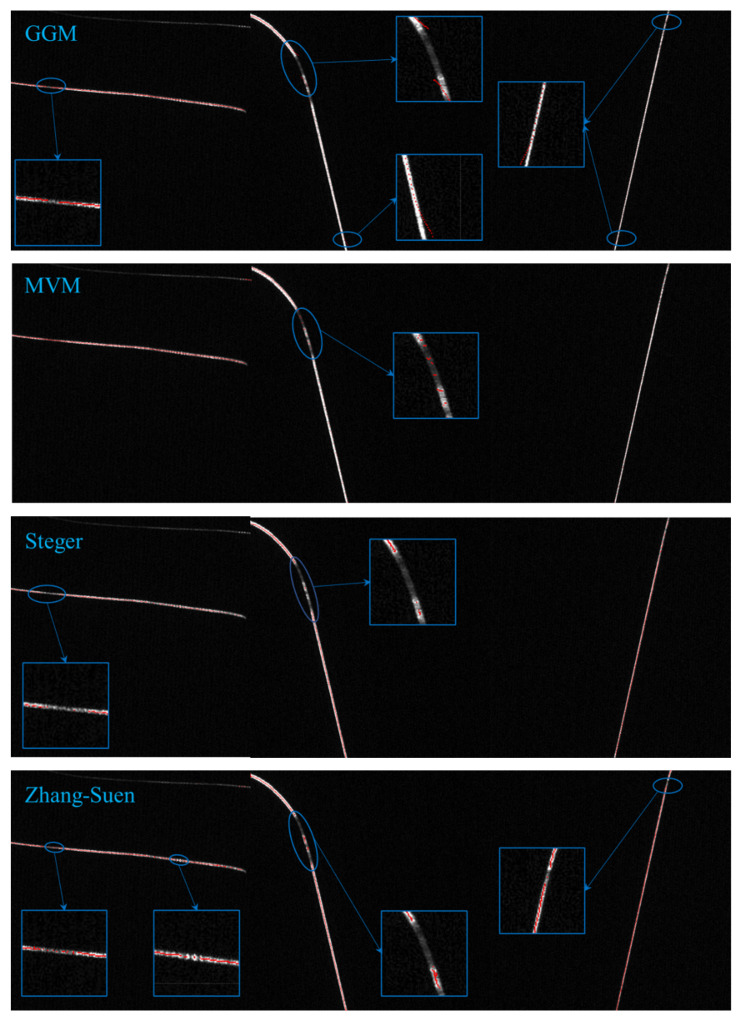

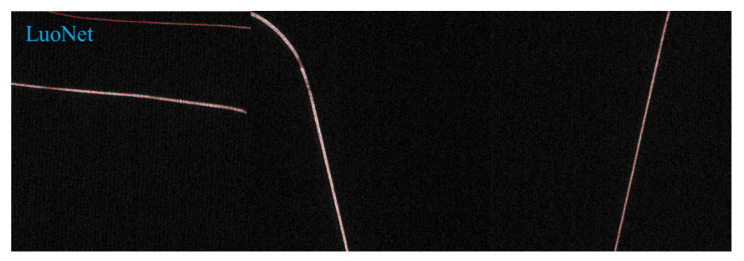
Comparison of extraction results by different methods.

In this paper, to test the extraction deep learning skeleton extraction network LuoNet and the subway wheel to extract the effectiveness of the laser curve, the test samples were selected under extreme conditions with an extremely low gray value, uneven gray distribution, and serious background interference. In order to improve the feasibility, transverse, longitudinal, and curved samples were selected for the experiment. We compared the common optical center extraction algorithm, as shown in Figure 12. From top to bottom is in accordance with the gray centroid method (GGM), the maximum value method (MVM), the Steger method, Zhang-Suen skeleton extraction (Zhang-Suen) [6], and deep learning method (LuoNet) extraction results. It can be seen that the traditional laser stripe extraction method is unable to do anything for the subway wheelset laser curve with uneven brightness. Due to the serious interference of ambient light and noise, the complete laser curve contour cannot be obtained. There is a discontinuity in the extraction of the light stripe center, and it is seriously missing in some places with low gray values. The gray center of gravity method has a higher requirement on the gray value distribution of the light stripe, and the effect is not very good for the situation that the gray value of the subway wheelset laser curve has a largely random distribution. As can be seen from the figure, the gray center of gravity method and extreme value method require the gray distribution of the stripe to be a Gaussian distribution. For the test samples we selected under extreme conditions, the gray center method could not find centers in some places with low gray values, and multiple centers appeared in some places. The extreme value method is more sensitive to noise, and the noise is judged in some areas, and the phenomenon of missing detection appears in places with low gray values. The extremum method only focuses on where the extremum occurs, and, in many places, there are multiple extremums, hence the emergence of multiple centers. The effect of Steger method is relatively good, but serious missed detection occurs in the area with low gray value, resulting in more discontinuities in the centerline. Steger method has a large amount of calculation and low efficiency, so it cannot extract the center of the strip quickly, and the inappropriate Gaussian kernel selection may lead to the distortion of image information. It is difficult to ensure that the result is a single pixel in the center of the light stripe extracted by the Zhang-Suen skeleton thinning method, and Burr and missing in some places. For the place with a low gray value and complete place, the traditional method is difficult to find the center by algorithm; the traditional method depends on the light intensity and gray level in the Gaussian distribution method. The gray level distribution is very irregular in terms of the conditions required to achieve good results. The LuoNet method of deep learning skeleton extraction proposed in this paper has a strong anti-interference ability, and the extraction results of the light stripe center are stable and clear.

In order to compare the accuracy of the laser curve extraction of a subway wheelset by different methods, we compared the accuracy and stability of a laser center extracted by different methods. Eighty subway wheels were chosen for laser curve images, and a precise comparison of the results was made to different extraction methods. Firstly, the light strip image is manually labeled, the mean distance between the extracted pixel coordinates and the manually labeled reference coordinates is calculated, and the obtained results are used as the error of the light strip extraction. The mean value of the distance extracted from the center of the stripe is defined as:

(6) $$\mathrm{MSE}=\frac{\sum_{n=1}^{N}\left[\sqrt{{\left(P_{i}^{n}-Q_{i}^{n}\right)}^{2}+{\left(P_{j}^{n}-Q_{i}^{n}\right)}^{2}}\right]}{N}$$

The N in the formula represents the rows or columns of the image $\left(P_{i}^{n},P_{j}^{n}\right)$ is the center coordinate of the nth row or column, and $\left(Q_{i}^{n},Q_{j}^{n}\right)$ is the manually labeled center coordinate. The smaller the MSE value is, the higher the precision of light stripe extraction is.

**Figure 13:**
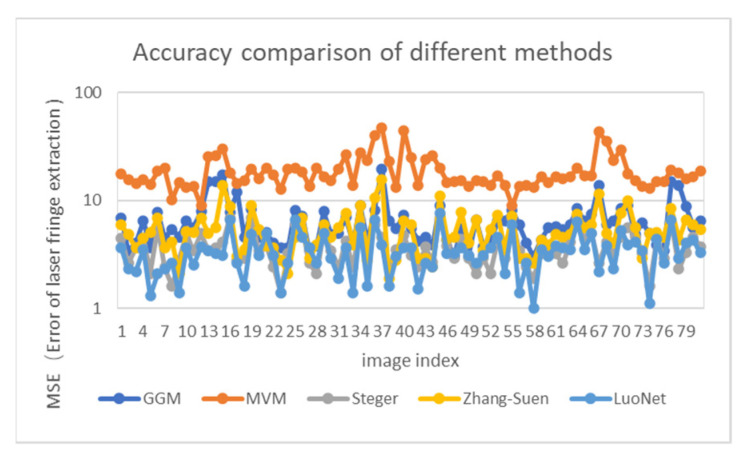
Precision comparison of different stripe center extraction methods.

Figure 13 shows the precision comparison results of extraction results by different extraction methods of the light stripe center. It can be seen that the error of the method proposed in this paper can be maintained at a relatively stable level (shown by the light blue line in the figure), and the average error is 3.28 pixels. Compared with other traditional methods, the extraction result is relatively stable.

**Figure 14:**
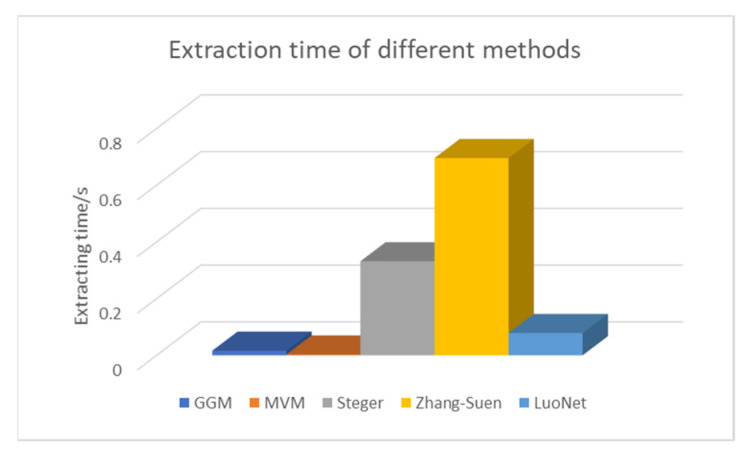
Comparison of extraction time of different light stripe centers.

Counting the time taken by different algorithms to extract the center of the light bar, the time diagram in Figure 14 can be drawn. The size of the test image is 512 * 512 pixels. It can be seen from the histogram that the GGM method represented by blue and the MVM represented by orange take the least time, but the extraction accuracy is poor. The Steger method represented by gray and the Zhang-Suen method represented by yellow require a large amount of computation and a high time complexity. Light blue represents the method proposed in this paper. The execution time of the algorithm proposed in this paper is 0.078s per image, which is less than the Steger method. The extraction accuracy and algorithm time complexity are considered comprehensively. Under the condition of ensuring the same accuracy, the algorithm proposed in this paper takes less time.

## 6. Conclusions

In this paper, a fast, robust, and accurate algorithm for extracting the centerline of a subway wheelset laser was proposed. The deep learning skeleton extraction method was adopted to design the LuoNet network according to the characteristics of the subway wheelset laser curve dataset, which can effectively remove the influence of ambient light and non-uniform reflected light on the light stripe extraction. The network adopts the cascade mode to realize the fusion of a high-dimensional feature graph and low-dimensional feature graph, and each cascade realizes the fusion of detail information and semantic information. The cascade layer number is successively reduced so as to minimize the loss of down-sampling. The attention YE Module proposed in this paper enhances the robustness of the algorithm and improves the accuracy of the algorithm. The algorithm proposed in this paper reaches 0.714 for F1_score, 0.781 for Miou, 3.28 pixels for MSE, and 0.078s for running time. Compared with different stripe center extraction algorithms, it is found that the proposed algorithm is significantly better than other algorithms. Through the experimental analysis, the proposed method can accurately identify the center of the stripe even when the gray value is low, the background interference is serious, and the quality of the stripe acquisition is poor. The actual environment of subway operation is a dark background, and the image acquisition process is disturbed by train jitter and ambient light, resulting in poor image quality. It is generally adopted to set up a multi-phase camera for shooting and select the image with the best quality as the image extracted by the laser. Obviously, this cost is relatively high. The method proposed by us can effectively overcome the problems caused by the poor quality of the laser stripe center extraction. The accuracy of the deep learning method largely depends on large-scale data training. The data collected in this paper is limited, so the accuracy does not reach our expected accuracy. In the next step, we will collect more data for different scenarios, so that the method can be applied to more complex conditions and extreme situations.

## Figures and Tables

**Table 1:** Comparison results of different networks.

Models	F1_Score	Precision	Recall	Miou
U-Net	0.6496	0.5419	0.8106	0.7134
SDRNet	0.6585	0.5573	0.8312	0.7372
PSPU_SkelNet	0.6787	0.5484	0.8902	0.7562
LuoNet	0.7140	0.6226	0.8345	0.7814

**Table 2:** YE Module ablation study.

Models	F1_Score	Precision	Recall	Miou
LuoNet (no attention)	0.6903	0.6231	0.7738	0.7616
LuoNet + SE	0.6949	0.6259	0.7810	0.7649
LuoNet + YE	0.7140	0.6226	0.8345	0.7814

**Table 3:** Models ablation study.

Models	F1_Score	Precision	Recall	Miou
LuoNet_A	0.6856	0.6188	0.7684	0.7602
LuoNet_B	0.6891	0.6178	0.7789	0.7623
LuoNet_C	0.6978	0.6268	0.7869	0.7674
LuoNet_AB	0.6969	0.6188	0.7976	0.7669
LuoNet_AC	0.6970	0.6375	0.7688	0.7670
LuoNet_BC	0.7033	0.6168	0.8181	0.7707
LuoNet (LuoNet_ABC)	0.7140	0.6226	0.8345	0.7814

## Data Availability

This work was supported by the China Fundamental Research Funds (6217010132) for the Central Universities, and the authors acknowledge them for their support. The authors also thank Southwest Jiaotong University NDT Research Center, Olympus NDT Joint Laboratory of Nondestructive Testing, and Chengdu Lead Science & Technology Co., Ltd. for their kind support in the experiment. Finally, thanks to Jiang Tianci from Waseda University in Japan and Li Chunjiang from Zhejiang University in China for their support of this paper.

## References

[1] Perona P., Malik J. (1990). Scale-space and edge detection using anisotropicdiffusion. IEEE Trans. Pattern Anal. Mach. Intell..

[2] Cai H., Yu Y., Huang Z., Si Q., Yu W. (2006). A new method of extracting the center of interference fringes based oncurve fitting. J. Optoelectron. Laser Tianjing China.

[3] Yang W. (2009). Research on the Method of Extracting the Center of Structured Light Stripe. Ph.D. Thesis.

[4] Hu B., Li D.H., Jin G. (2002). Detection method of stripe center of structured light based on direction template. Comput. Eng. Appl..

[5] Steger C. (1998). An Unbiased Detector of Curvilinear Structures. IEEE Trans. Pattern Anal. Mach. Intell..

[6] Zhang T.Y., Suen C.Y. (1984). A fast parallel algorithm for thinning digital patterns. Commun. ACM.

[7] Xu N. (2007). Research on Image Processing Method of Line Structured Light Stripe. Ph.D. Thesis.

[8] Wang S.C., Han Q., Wang H. (2019). Extraction method of laser stripe center of rail profile in driving environment. Acta Opt. Sin..

[9] Yang K., Luo S., Wang Y., Gao X., Jiang T., Li C., Zhao Y. (2021). Laser curve extraction of train wheelset based on UNet. Nondestruct. Test..

[10] Demir I., Hahn C., Leonard K., Morin G., Rahbani D., Panotopoulou A., Kortylewski A. Skeleton 2019: Dataset and challenge on deep learning for geometric shape understanding. Proceedings of the IEEE/CVF Conference on Computer Vision and Pattern Recognition Workshops.

[11] Jiang N., Zhang Y., Luo D., Liu C., Zhou Y., Han Z. Feature hourglass network for skeleton detection. Proceedings of the 2019 IEEE/CVF Conference on Computer Vision and Pattern Recognition Workshops (CVPRW).

[12] Nathan S., Kansal P. SkeletonNet: Shape pixel to skeleton pixel. Proceedings of the 2019 IEEE/CVF Conference on Computer Vision and Pattern Recognition Workshops (CVPRW).

[13] Panichev O., Voloshyna A. U-net based convolutional neural network for skeleton extraction. Proceedings of the 2019 IEEE/CVF Conference on Computer Vision and Pattern Recognition Workshops (CVPRW).

[14] Dey S. Subpixel dense refinement network for skeletonization. Proceedings of the 2020 IEEE/CVF Conference on Computer Vision and Pattern Recognition Workshops (CVPRW).

[15] Hu J., Shen L., Albanie S., Sun G., Wu E. (2017). Squeeze-and-Excitation Networks. IEEE Trans. Pattern Anal. Mach. Intell..

[16] Ronneberger O., Fischer P., Brox T. U-Net: Convolutional Networks for Biomedical Image Segmentation. Proceedings of the International Conference on Medical Image Computing and Computer-Assisted Intervention.

[17] Atienza R. Pyramid U-network for skeleton extraction from shape points. Proceedings of the 2019 IEEE/CVF Conference on Computer Vision and Pattern Recognition Workshops (CVPRW).

